# Pro-inflammatory cytokines and lipopolysaccharide induce changes in cell morphology, and upregulation of ERK1/2, iNOS and sPLA_2_-IIA expression in astrocytes and microglia

**DOI:** 10.1186/1742-2094-8-121

**Published:** 2011-09-24

**Authors:** Wenwen Sheng, Yijia Zong, Arwa Mohammad, Deepa Ajit, Jiankun Cui, Dongdong Han, Jennifer L Hamilton, Agnes Simonyi, Albert Y Sun, Zezong Gu, Jau-Shyong Hong, Gary A Weisman, Grace Y Sun

**Affiliations:** 1 Department of Biochemistry, University of Missouri, Columbia, Missouri 65211, USA; 2 Interdisciplinary Neuroscience Program, University of Missouri, Columbia, Missouri 65211, USA; 3 Department of Biological Sciences, University of Missouri, Columbia, Missouri 65211, USA; 4 Christopher S. Bond Life Sciences Center, University of Missouri, Columbia, Missouri 65211, USA; 5 Department of Pathology and Anatomical Sciences, University of Missouri Medical School, Columbia, Missouri 65212, USA; 6 Center for Translational Neurosciences, University of Missouri Medical School, Columbia, Missouri 65212, USA; 7 Laboratory of Toxicology and Pharmacology, National Institute of Environmental Health Sciences, National Institutes of Health, Research Triangle Park, North Carolina 27709, USA

**Keywords:** BV-2, HAPI, DITNC, primary astrocytes, primary microglial cells, sPLA2-IIA, iNOS, ERK1/2, filopodia

## Abstract

**Background:**

Activation of glial cells, including astrocytes and microglia, has been implicated in the inflammatory responses underlying brain injury and neurodegenerative diseases including Alzheimer's and Parkinson's diseases. Although cultured astrocytes and microglia are capable of responding to pro-inflammatory cytokines and lipopolysaccharide (LPS) in the induction and release of inflammatory factors, no detailed analysis has been carried out to compare the induction of iNOS and sPLA2-IIA. In this study, we investigated the effects of cytokines (TNF-alpha, IL-1beta, and IFN-gamma) and LPS + IFN-gamma to induce temporal changes in cell morphology and induction of p-ERK1/2, iNOS and sPLA_2_-IIA expression in immortalized rat (HAPI) and mouse (BV-2) microglial cells, immortalized rat astrocytes (DITNC), and primary microglia and astrocytes.

**Methods/Results:**

Cytokines (TNF-alpha, IL-1beta, and IFN-gamma) and LPS + IFN-gamma induced a time-dependent increase in fine processes (filopodia) in microglial cells but not in astrocytes. Filopodia production was attributed to IFN-gamma and was dependent on ERK1/2 activation. Cytokines induced an early (15 min) and a delayed phase (1 ~ 4 h) increase in p-ERK1/2 expression in microglial cells, and the delayed phase increase corresponded to the increase in filopodia production. In general, microglial cells are more active in responding to cytokines and LPS than astrocytes in the induction of NO. Although IFN-gamma and LPS could individually induce NO, additive production was observed when IFN-gamma was added together with LPS. On the other hand, while TNF-alpha, IL-1beta, and LPS could individually induce sPLA_2_-IIA mRNA and protein expression, this induction process does not require IFN-gamma. Interestingly, neither rat immortalized nor primary microglial cells were capable of responding to cytokines and LPS in the induction of sPLA2-IIA expression.

**Conclusion:**

These results demonstrated the utility of BV-2 and HAPI cells as models for investigation on cytokine and LPS induction of iNOS, and DITNC astrocytes for induction of sPLA2-IIA. In addition, results further demonstrated that cytokine-induced sPLA2-IIA is attributed mainly to astrocytes and not microglial cells.

## Background

Activation of glial cells, including astrocytes and microglial cells, has been implicated in the inflammatory responses in brain injury and in neurological diseases such as Alzheimer's disease, Parkinson's disease and stroke [1-5]. Astrocytes and microglia are two distinct types of glial cells in the central nervous system. Despite obvious differences in morphology and functional properties, they are regarded as immune active cells and in some instances, they share common innate immune responses. For example, both astrocytes and microglial cells have been shown to respond to pro-inflammatory cytokines and lipopolysaccharide (LPS) in the induction of iNOS as well as other inflammatory factors [6-11]. However, difficulties in obtaining pure and large quantities of astrocytes and microglial cells in primary cultures have led to studies using immortalized cells. In recent years, immortalized microglial cells, such as the murine derived BV-2 cells, have been extensively used as cell models to elucidate signaling pathways and responses to pro-inflammatory cytokines and LPS [9,12].

The secretory phospholipase A2 (sPLA2) family is comprised of a group of low molecular mass enzymes [13], and sPLA_2_-IIA has long been regarded as an inflammatory protein associated with infection and cardiovascular diseases [14,15]. In the central nervous system, upregulation of sPLA2-IIA has been shown in rat brain in response to focal cerebral ischemic injury [16,17], as well as in the human Alzheimer brain as compared with age-matched controls [18]. Upregulation of sPLA2-IIA expression is also found in the rat model for spinal cord injury [19,20]. Studies with cultured cells have shown the ability for astrocytes to induce sPLA2-IIA in response to pro-inflammatory cytokines [21-23]. However, whether cytokines and LPS can induce sPLA2-IIA expression in activated microglial cells has not been investigated in detail. Due to a point-shift mutation in many murine species [24], studies to investigate sPLA2-IIA expression have been limited to astrocytes and microglial cells derived from rat brains. The rat-derived Highly Aggressive Proliferating Immortalized microglial cells (HAPI) were derived from mixed glial cultures in rat brains [25]. While the HAPI cells show many similarities to BV-2 cells, there are obvious differences in inflammatory responses comparing HAPI, BV-2, and primary microglial cells [26]. In this study, the murine BV-2 cells, rat HAPI microglial cells, and the middle T-antigen derived immortalized astrocytes (type II) from rat diencephalon (DITNC) together with primary astrocytes and microglial cells were used to examine induction of iNOS and sPLA_2_-IIA expression by pro-inflammatory cytokines (TNFα, IL-1β, and IFNγ) and by LPS+IFNγ.

## Methods

### Materials

Dulbecco's modified Eagle's medium (DMEM), penicillin, streptomycin, 0.05% (w/v) trypsin/EDTA, and phosphate-buffered saline (PBS) were obtained from GIBCO-BRL (Gaithersburg, MD, USA). Cytokines (TNFα, IL-1β, and IFNγ) were purchased from R & D Systems (Minneapolis, MN, USA). Lipopolysaccharide (LPS) (rough strains) from *Escherichia coli *F583 (Rd mutant) were purchased from Sigma-Aldrich (St. Louis, MO, USA). Fetal bovine serum was from Atlanta Biologicals (Lawrenceville, GA, USA). Methylthiazolyldiphenyl-tetrazolium bromide (MTT) was from Sigma-Aldrich (St. Louis, MO, USA). Antibodies for Western blot are: (1) sPLA2-IIA human, rabbit polyclonal antibody (BioVendor, Candler, NC); (2) goat anti-rabbit IgG- horseradish peroxidase (Santa Cruz Biotechnology, Santa Cruz, CA); and (3) monoclonal anti-β-actin peroxidase (Sigma - Aldrich, St. Louis, MO). Antibodies for immunohistochemistry are: (1) anti-sPLA2-IIA polyclonal antiserum (Cayman Chemical, Ann Arbor, MI); (2) anti-GFAP monoclonal antibody for astrocytes (Millipore, Billerica, MA); (3) CD11b antibody (Abcam Inc., cat # ab63317 Inc, Cambridge, MA); (4) fluorescein isothiocyanate (FITC)-labeled goat anti-mouse and Texas red-labeled goat anti-rabbit secondary antibody (Santa Cruz Biotechnology, Santa Cruz, CA); and (5) Rhodamine-phalloidin (Molecular Probes, Eugene, OR) for F-actin.

### Cell culture preparations and morphological examination

Preparations of primary astrocytes and microglial cells involved pregnant Sprague-Dawley rats and C57BL/6 mice (Harlan, IN, USA) and 1-3 day-old pubs. All animal care and experimental protocol with post-natal pups were carried out in accordance with NIH guidelines and with the University of Missouri Animal Care and Use Committee (protocol #6728).

The immortalized mouse microglial cells (BV-2) were originally obtained from Dr. R. Donato (University of Perugia, Italy) and cultured as described previously [9]. Briefly, cells were cultured in 75 cm^2 ^flasks with DMEM (high glucose) supplemented with 5% FBS containing 100 units/ml penicillin and 100 μg/ml streptomycin, and maintained in 5% CO_2 _incubator at 37°C. For subculture, cells were removed from the culture flask with a scraper, re-suspended in the culture medium and subcultured in 12-well (0.4 × 10^6^) or 6-well (1.0 × 10^6^) plates for experiments. In some experiments, cells were cultured in cover slips and used for immunostaining.

The immortalized rat microglial cell line HAPI was a generous gift from Dr. J. Hong (Laboratory of Toxicology and Pharmacology, National Institute of Environmental Health Sciences, National Institute of Health, Research Triangle Park, NC). The immortalized rat astrocytes, DITNC, were obtained from ATCC (Rockville, MD, USA). Both HAPI and DITNC cells were cultured in DMEM (high glucose), 10% FBS, 100 units/ml penicillin, and 100 μg/ml streptomycin and maintained in 5% CO2 at 37°C. To harvest HAPI microglia and DITNC astrocytes, cells were treated with 0.05% trypsin/EDTA for 2 minutes at 37°C, and centrifuged at 125 *g *for 10 min. The cell pellets were re-suspended in culture medium. Cell concentration was determined by counting cells with a hemocytometer. Cells were subcultured in 12-well (0.4 × 10^6^) or 6-well (1.0 × 10^6^) plates for experiments.

Primary astrocytes were prepared from the cerebral cortices of 1- 3 day-old Sprague-Dawley rats (Harlan, IN, USA) as described by McCarthy and deVellis [27] with slight modifications [28]. Briefly, cerebral cortices were dissected and meninges removed. The tissues were minced and suspended in 10 volumes 0.05% (w/v) trypsin/EDTA and incubated for 10 min at 37°C. The cell suspension was passed through a 14-gauge needle 5 times, and then filtered through 85 mm nylon mesh. The filtrate was sedimented by centrifugation at 200 *g *for 5 min and re-suspended in 10% FBS in DMEM containing 100 units/ml penicillin and 100 μg/ml streptomycin. Finally, cells were transferred to 75 cm^2 ^culture flasks and fresh medium was changed the next day and then every 2 days afterwards. When cells became confluent, normally within 7-9 days, flasks were shaken at 200 rpm on an orbital shaker (Fisher Scientific, St. Louis, MO) for 4 h at room temperature to remove microglial cells. After shaking, cells were rinsed three times with phosphate-buffered saline (PBS), suspended in trypsin-containing solution as above, and subcultured in 12-well plates for Griess reaction experiment and 6-well plates for Western blot analysis. These cultures contained over 95% astrocytes, as determined by immunostaining for glial fibrillary acidic protein (GFAP). For immunohistochemistry experiments, astrocytes were cultured on Poly-L-Lysine Coated Glass Coverslips (12 mm Round No. 1 German Glass) (BD Biosciences, San Jose, CA). Cells were starved for 4 h prior to experimentation in serum free DMEM medium and followed by treatments with different conditions as described.

For preparation of primary microglial cells, rat (Sprague-Dawley) or mouse (C57BL/6) pups less than 4 days of age were used. The protocol was similar to that used for preparation of primary astrocytes. Briefly, after removing the meninges, brain tissue was minced into small pieces and trypsinized by incubating tissue at 37°C for 20 min. Brain tissue was triturated with a pipet to further dissociate clumps and filtered with a 70 μm cell strainer. Cells were centrifuged at 1,200 rpm for 5 min at 4°C, and pellet was suspended in 30 ml of complete medium containing DMEM with high glucose, 10% FBS, OPI (1 mM oxaloacetate, 0.45 mM pyruvate, and 0.2 U/ml insulin), and GM-CSF (0.5 ng/ml) to enhance proliferation of microglia. The cell suspension was added to 75 cm^2 ^flasks (about 6 brains per flask of 30 ml). Cells were incubated in flasks until confluent for 7-10 days. Microglial cells were separated from astrocytes and oligodendrocytes by shaking the flasks in a rotary platform in a 37°C incubator at 200 rpm overnight. The supernatant, which was enriched with microglial cells, was then removed and centrifuged at 1200 rpm for 45 min. The microglia population was established by immunostaining with CD11b antibody. Purity for these microglial cells was determined to be around 95% (data not shown). The cells were plated for experiments using complete media without the GM-CSF.

In all experiments, cells were serum starved for 4 h prior to adding cytokines and LPS. Cell morphology was observed by using a phase contrast Nikon DIAPHOT 300 microscope attached with a CCD cool camera linked to MagnaFire 2.1C software for image processing. Representative bright field pictures were obtained using a 20× objective lens.

### Measurement of NO

Our previous studies demonstrated that NO production in glial cells was mainly due to the induction of iNOS [9]. Therefore, measurement of NO was used to represent the induction process. NO released from cells was converted to nitrite in the culture medium, which was determined using the Griess reagent. In this study, cells were cultured in DMEM without phenol red. After treating cells with cytokines and LPS, aliquots (200 μl) of culture medium were transferred to test tubes and incubated with 100 μl of the reagent A (1% (w/v) sulfanilamide in 5% phosphoric acid, Sigma) for 10 minutes at room temperature in the dark. This was followed by incubation with 100 μl of reagent B (0.1%, w/v, N-1-napthylethylenediamine dihydrochloride, Sigma) for 10 minutes at room temperature in the dark. After mixing, 100 μl of the purple/magenta solution was transferred to a 96-well plate and the absorbance at 543 nm was measured within 30 minutes in a plate reader. The dilution series of sodium nitrite (0-100 μM) was used to generate the nitrite standard reference curve.

### Western blot analysis

After treating cells with cytokines and LPS, cells were washed twice with ice-cold phosphate-buffered saline and harvested in lysis buffer containing 50 mM Tris-HCl (pH 7.4), 1 mM EDTA, 100 mM NaCl, 0.1% SDS, 1 mM PMSF, 1 mM sodium orthovanadate, 1 μg/ml leupeptin, 1 μg/ml pepstatin, and 10 μg/ml aprotinin. The extract was centrifuged at 10,000 × g for 15 minutes at 4°C in order to get rid of cell debris. Protein concentration was determined by using a BCA protein assay kit (Pierce Biotechnology, Rockford, IL) according to the manufacturer's instructions. Equivalent amounts of protein (25 μg) for each sample were resolved in 12% Tricine-SDS-PAGE at 120 V in duplicates. After electrophoresis, proteins were transferred to 0.2 μm PVDF membranes at 250 mA for 2 h. Membranes were incubated in Tris-buffered saline, pH 7.4 (TBS) with 0.1% Tween 20 (TBS-T) containing 5% non-fat milk for 1 h at room temperature. The blots were then incubated with sPLA2-IIA polyclonal antibody (1:2500; BioVendor, Candler, NC) overnight at 4°C. After washing with TBS-T, blots were incubated with goat anti-rabbit IgG- horseradish peroxidase (1:5000; Santa Cruz Biotechnology, Santa Cruz, CA) for 1 h at room temperature. The blots were then washed three times with TBS-T. Immunolabeling was detected by chemiluminescence (SuperSignal West Pico, Pierce, Rockford, IL). For loading control, the blots were reacted with monoclonal anti-β-actin peroxidase (1:30,000, Sigma - Aldrich, St. Louis, MO). For quantification, blots were scanned and the intensity of protein bands was measured as optical density using the Quantity One program (BioRad, Hercules, CA). sPLA2-IIA bands were detected at 15 kDa. Ratios of sPLA2-IIA to β-actin were calculated for each sample.

### Immunohistochemistry

DITNC cells and primary astrocytes were plated onto poly-L-lysine coated glass coverslips. After treatments, cells were fixed in 4% paraformaldehyde in PBS (pH 7.4) for 15 min at room temperature. After washing three times with PBS, samples were incubated for 10 min with PBS containing 0.5% Triton-X-100. Nonspecific binding of antibodies was blocked by 5% normal goat serum (NGS) for 1 h at room temperature. Cells were then incubated overnight at 4°C in 0.5% NGS with anti-sPLA2-IIA polyclonal antiserum (1:50, Cayman Chemical, Ann Arbor, MI), anti-GFAP monoclonal antibody for astrocytes (1:50, Millipore, Billerica, MA), or anti-CD11b antibody for microglial cells (1:100, Abcam Inc, Cambridge, MA). The cells were washed with PBS and incubated for 1 h at room temperature with fluorescein isothiocyanate (FITC)-labeled goat anti-mouse and Texas red-labeled goat anti-rabbit secondary antibody (1:300, Santa Cruz Biotechnology, Santa Cruz, CA), and finally washed again with PBS. Cells were incubated for 10 min with Hoechst 33342 (1: 1000, Invitrogen, Carlsbad, CA) as a counter-stain for nuclei. Cover-slips were then mounted onto microscope slides and fluorescent intensity measurements were performed at room temperature using the Olympus X-41 fluorescence microscope and 40× objective lens.

For immunofluorescence staining of F-actin, BV-2 cells in cover-slips were fixed with 4% paraformaldehyde for 20 min and permeabilized by 0.1% Triton X-100 in PBS for 10 min. Non-specific binding was blocked with 5% normal goat serum (NGS) in PBS at room temperature for 30 min. Cells were then incubated in rhodamine-phalloidin (Molecular Probes, Eugene, OR), diluted 1:100 in PBS for 30 min, and then mounted onto microscope slides and examined using the Leica DMI4000 epifluorescence microscope with 40× objective lens.

### RT-PCR

After treating cells with cytokines and LPS, total RNA was isolated from cells using the TRIZOL reagent (Sigma-Aldrich, MO, USA). The RNA quality and concentration was evaluated by Nanodrop ND-1000 spectrophotometry (NanoDrop Technologies, Wilmington, DE). OD_260 _was used for the concentration while OD_260_/OD_280 _and OD_260_/OD_230 _were used to evaluate the quality, usually ~1.8-2.2. Total RNA (0.5 μg) was used for reverse transcription to cDNA with oligo dT primers by means of the Advantage RT-for-PCR Kit (Takara Bio, Mountain View, CA) according to the manufacturer's instructions. The volume of cDNA used was 10 μl (from 50 ng RNA). Amplification was carried out in an automated thermal cycler (Eppendorf, Hauppauge, NY) with a 3-min denaturation step at 94°C, followed by 25 cycles including 45 sec at 94°C, 30 sec at 59.5°C, and 30 sec at 72°C. All PCR amplifications were submitted to a final 10-min step at 72°C. Amplified samples were separated on a 2% agarose gel containing ethidium bromide in TAE buffer. After electrophoresis, the gel was viewed by the Kodak electrophoresis documentation and analysis system (Kodak, Rochester, NY). Primers for rat sPLA_2_IIA are: sense 5'-CATGGCCTTTGGCTCAATTCAGGT-3'; antisense 5'-ACAGTCATGAGTCACACAGCACCA-3'; and rat G3PDH sense 5'-TGAAGGTCGGTGTCAACGGATTTGGC-3'; antisense 5'-CATGTAGGCCATGAGGTCCACCAC-3' was used as a control.

### Quantitation of filopodia

For study to quantitate filopodia in BV-2 microglia, cells were cultured in 35 mm dish until 80% confluency. Cells were serum starved for 4 h prior to treatment with cytokines and LPS. Since thin processes (filopodia) started to appear after cytokine treatment by 2 h, a 4 h exposure time was used for quantitaion of filopodia. In each treatment condition, cells were observed under the phase contrast Nikon DIAPHOT 300 microscope and three fields with comparable dell densities were chosen. In each field, the total number of cells, as well as cells containing filopodia (processes more than 2 mm), were counted. Results are expressed as % of filopodia-containing cells against the total.

### Assessing cell viability

Cell viability was determined using the MTT (3-(4, 5-Dimethylthiazol-2-yl) -2, 5-diphenyltetrazolium bromide) assay protocol. Briefly, cells cultured in 12-well plates were treated with cytokines and LPS. After treatment, the medium was removed and 1 ml of MTT reagent (0.5 mg/ml) in serum free DMEM was added into each well. Cells were incubated for 4 h at 37°C, and after dissolving the formazan dye with DMSO, absorption was read at 540 nm.

### Statistical analysis

Results are analyzed by one-way ANOVA followed by Dunett's multiple comparison tests, or two-way ANOVA (V4.00; GraphPad Prism Software Inc., San Diego, CA). Differences with p < 0.05 are considered significant.

## Results

### Cytokines and LPS induce morphological changes in microglial cells and astrocytes

**Figure 1:**
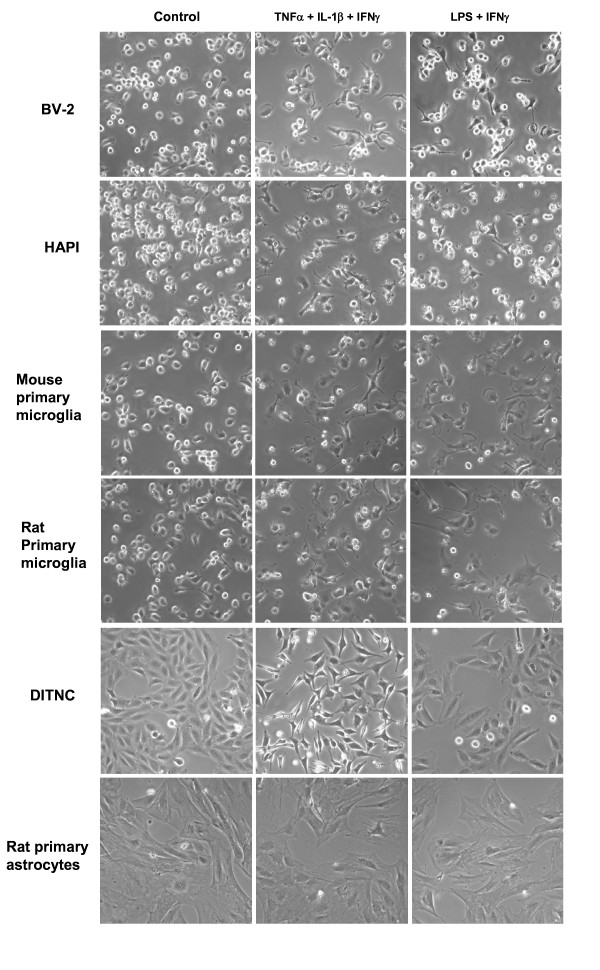
**Cytokines (mixture of TNFα, IL-1β, IFNγ) or LPS + IFNγ alter morphology of microglial cells and astrocytes**. Cells were cultured in 12-well plates and serum starved for 4 h before treatment with the three cytokine mixture (3 cyt) containing TNFα, IL-1β, and IFNγ at 10 ng/ml each respectively, or LPS (100 ng/ml) + IFNγ (10 ng/ml) for 24 h. Cell morphology was obtained by taking bright field pictures with an inverted Nikon microscope (20×) at 24 h with and without (Control) treatment with cytokines and LPS. Photomicrographs are representative pictures depicting BV-2 (murine) and HAPI (rat) microglial cells, mouse and rat primary microglial cells, and rat immortalized (DITNC) astrocytes and rat primary astrocytes.

Based on preliminary study and results in Table 1 treating BV-2 microglial cells with a mixture of three cytokines (TNFα, IL-1β, and IFNγ, at 10 ng/ml each) or LPS (100 ng/ml) + IFNγ (10 ng/ml) produce high levels of NO. These conditions were used to examine cell morphology and viability in different glial cell types. In this study, cells were cultured to 90% confluency, and at 4 h prior to treatment with cytokines and LPS, serum was removed from the cultures and replaced with DMEM. Bright field pictures depicting cell morphology with or without cytokine and LPS treatments were obtained at 24 h using the inverted Nikon microscope. As shown in Figure 1, control BV-2 and HAPI cells (incubated for 24 h under serum free condition) are mostly round with bright refringency and small dark nuclei; whereas, cytokine and LPS treatments for 24 h caused cells to become ramified and some are star shaped with short thick processes. Removal of serum retarded cell growth but did not cause morphological changes (data not shown). Control and treated primary mouse and rat microglial cells (derived from primary astrocytes) show similar morphology and responses as compared to immortalized microglial cells (Figure 1).

**Table 1:** NO production by cytokines and LPS in different glial cell types

*Cell types* *(number)*	*Con*	*TNFα+* *IL1β*	*IFNγ*	*TNFα+IL1β* *+ IFNγ*	*LPS*	*LPS+* *IFNγ*
BV-2 (1 × 10^5^)	-	-	31.0 ± 0.5	69.5 ± 2.1	12.3 ± 2.4	79.1 ± 2.5
HAPI (1 × 10^5^)	-	-	17.2 ± 0.5	22.4 ± 0.5	12.9 ± 0.9	39.9 ± 0.7
DITNC (1 × 10^5^)	-	-	-	9.0 ± 0.2	-	-
RPA (3 × 10^5^)	-	-	-	4.6 ± 1.2	-	-

Results are expressed as NO in μM and are means ± SD from three experiments.

RPA, rat primary astrocytes

DITNC astrocytes are triangular shape with spindle-like features, and after treatment with the three cytokine mixture, they became dark with a bright refringency, but did not show obvious morphological changes as compared with microglial cells (Figure 1). Primary rat astrocytes are larger flat cells with irregular shape, and they do not show obvious morphological changes after exposure to cytokines and LPS (Figure 1).

**Figure 2:**
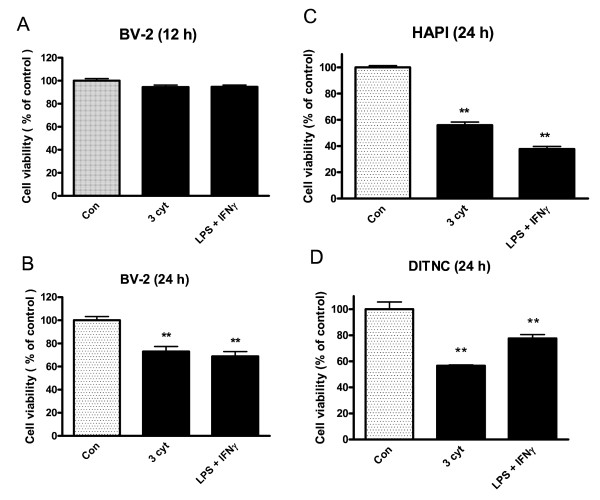
**Assay for cell viability after treating microglial cells and astrocytes with cytokine mixture or LPS + IFNγ**. BV-2 and HAPI microglial cells and DITNC astrocytes cultured in 12-well plate were serum starved for 4 h followed by exposure to the three cytokine mixture (3 cyt) or LPS + IFNγ for the respective time indicated. Cell lysates were obtained for MTT assay. Results are mean ± SD from n = 3. **p < 0.01 vs. control (Con).

We determined cell viability at 24 h after treating BV-2, HAPI, and DITNC astrocytes with cytokines and LPS + INFγ using the MTT assay protocol. In BV-2 cells, no change in MTT values was observed after exposure with the three cytokine mixture or LPS + INFγ for 12 h (Figure 2A). However, there are obvious decreases in MTT values in BV-2, HAPI, and DITNC cells at 24 h after exposure to cytokine and LPS + INFγ (Figures 2B-2D).

### Cytokines and LPS elicit different temporal profile for p-ERK1/2 between BV-2 microglia and DITNC astrocytes

**Figure 3:**
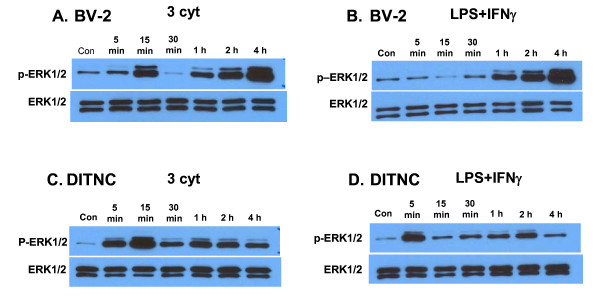
**Time course for p-ERK1/2 and total ERK1/2 expression after treating BV-2 and DITNC cells to the three cytokine mixture or LPS + IFNγ. BV-2 and DITNC cells were serum starved for 4 h and exposed to 3 cyt or LPS + IFNγ**. Cells lysates were obtained at indicated times and subjected to Western blot. Results are representative blots from three independent experiments.

Although earlier studies had demonstrated involvement of the MEK1/2-ERK1/2 pathway in cytokine-induced sPLA2 in DITNC astrocytes [21] and iNOS in BV-2 cells [9], a time course study to compare p-ERK1/2 activation in these two cell types was not carried out. As shown in Figure 3A, exposure of BV-2 cells to the three cytokine mixture showed a biphasic increase in p-ERK1/2; first a transient earlier phase peaking at 15 min, and then a second phase increase from 1 to 4 h. Exposure of BV-2 cells to LPS + IFNγ did not show the early phase increase, but a similar second phase of increase from 1 to 4 h (Figure 3B). Exposure of DITNC astrocytes to the three cytokine mixture indicated an early phase increase at 15 min and a second increase at 1 h (Figure 3C). Exposure of DITNC astrocytes to LPS + IFNγ also showed an early phase increase in pERK1/2 at 5 min and a subsequent phase at 2 h (Figure 3D). Unlike the BV-2 cells, DITNC astrocytes did not show a dramatic increase in p-ERK1/2 between 1 to 4 h.

### Cytokines induce time-dependent cytoskeletal changes and increase in filopodia in microglial cells

**Figure 4:**
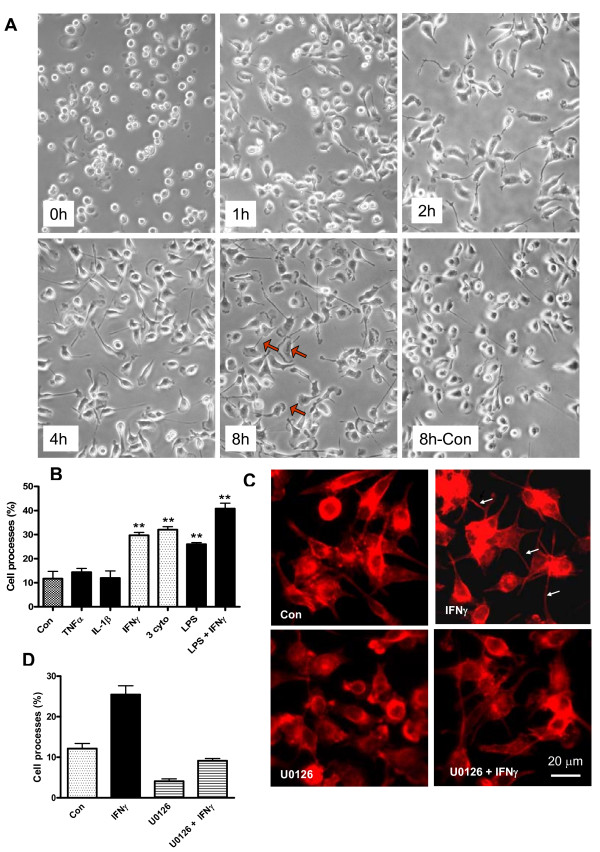
**Cytokines induce filopodia production in BV-2 cells**. (A) Time course for cytokine-induced filopodia formation in BV-2 cells. Representative bright field photomicrographs were taken with an inverted Nikon microscope (20×). Red arrows show processes with a fan-like ending. (B) Counting cells containing filopodia at 4 h after exposure to individual cytokines, LPS or combination as indicated. Results are expressed as % of filopodia cells versus total cell numbers (see Methods). Results are mean ± SEM from 4 independent experiments. Results are analyzed by one-way ANOVA followed by Dunnett's multiple comparison test, **p < 0.01 vs. control. (C) Staining F-actin in BV-2 cells after treatment with IFNγ and/or MEK1/2 inhibitor U0126. BV-2 cells were cultured in coverslip and serum starved for 4 h. Cells were pretreated with U0126 for 30 min prior to exposure to IFNγ for 4 h. Cells were then fixed with 4% paraformaldehyde and permeabilized by 0.1% Triton X-100 in PBS as described in text. After blocking non-specific binding with 5% normal goat serum (NGS), cells were incubated in rhodamine-phalloidin (1:100) and then mounted onto microscope slides and examined using the Leica DMI4000 automatic epifluorescence microscope with 40× objective lens. Space bar: 20 μm. White arrows denote filopodia. (D) Bar graph representing filopodia-containing cells after incubation with/without IFNγ, U0126, and U0126 + IFNγ. Two-way ANOVA revealed a significant interaction (p = 0.009) between U0126 and IFNγ, and a significant effect of U0126 (p < 0.0001), and IFNγ (p < 0.0001).

We further examined the time course for morphological changes after exposing BV-2 cells to the three cytokine mixture. As shown in Figure 4A, exposure of cytokines to BV-2 cells caused the cells to become elongated with protrusion of short fine processes (filopodia) as early as 1 h. The filopodia continued to become elongated with time and by 8 h, nearly all cells showed filopodia and some have flat pancake-like structures with ruffled edges at the end (red arrows). With increasing time, filopodia started to disappear between 12 to 16 h leaving cells with stout processes as shown in Figure 1. HAPI cells show a similar time-dependent increase in filopodia as in BV-2 cells (data not shown).

Since filopodia were produced after exposing BV-2 cells to the three cytokine mixture (TNFα, IL-1β, and IFNγ) and LPS + IFNγ, we further examined filopodia formation by treating cells with individual cytokines and LPS. As shown in Figure 4B, among the three cytokines tested, filopodia were only induced by IFNγ. Although LPS alone could also induce filopodia formation, the addition of IFNγ further enhanced formation of these processes (Figure 4B).

Since ERK activation has been shown to participate in IFNγ-mediated signaling pathways and cell migration [29,30], we tested whether p-ERK1/2 plays a role in IFNγ-induced filopodia formation. In this experiment, BV-2 cells were cultured in cover slips and serum starved for 4 h. After preincubated for 30 min with U0126 (10 μM), a specific inhibitor for MEK/ERK, they were exposure to IFNγ for 4 h. After the 4 h treatment, cells were subsequently stained for F-actin with rhodamine-phalloidin, a high-affinity F-actin probe. As shown in Figure 4C and 4D, exposing cells to IFNγ for 4 h resulted in formation of filopodia (white arrows). Treatment of cells with 10 μM of U0126 caused the cells to become round, and pretreatment of U0126 prior to exposure to IFNγ completely abrogated the formation of filopodia induced by IFNγ.

### Cytokines and LPS induce NO production in different glial cell types

**Figure 5:**
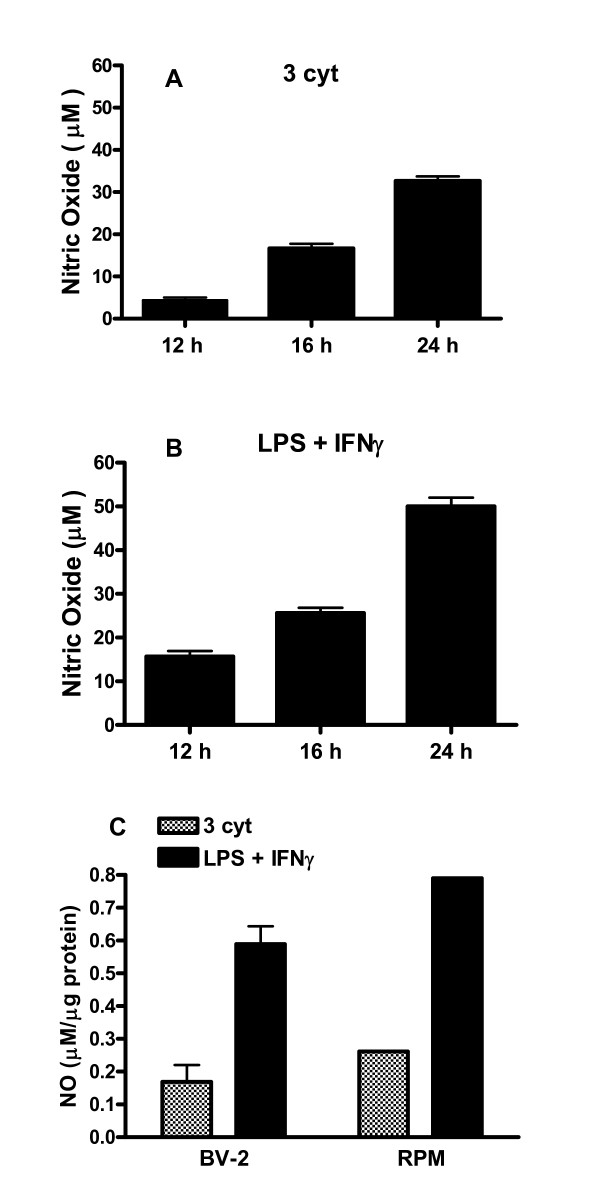
**Cytokines or LPS + IFNγ induce NO production in different glial cell types**. (A and B) BV-2 cells were serum starved in phenol red free DMEM for 4 h prior to treatment with the three-cytokine mixture (A) or LPS + IFNγ(B). At the respective time points, culture media were collected for determination of NO using the Greiss reaction protocol as described in the text. Data are mean ± SD from three independent experiments. **p < 0.01 vs. values at 12 h. One-way ANOVA, Dunnett's multiple comparison tests. (C) Comparing NO production between BV-2 microglial cells and rat primary microglial cells (RPM) based on protein in the dish. Results on RPM preparation have been repeated.

Our earlier studies demonstrated that NO production upon exposure of BV-2 cells to IFNγ and LPS is due mainly to induction of iNOS expression [9]. In this study, a time course experiment to compare NO production due to the three cytokine mixture and LPS + IFNγ indicated a detectable increase from 12 h to 24 h (Figure 5A and 5B). A similar time course for NO production was observed with the HAPI cells. In a subsequent experiment, induction of NO by individual cytokines and LPS was examined in BV-2, HAPI, DITNC and primary rat astrocytes after 24 h exposure. Similar to studies observed with BV-2 cells [9], TNFα + IL-1β could not induce NO in any of the cell types tested (Table 1). However, IFNγ alone can induce NO in both BV-2 and HAPI microglial cells and IFNγ enhanced NO production induced by LPS (Table 1). Under similar conditions, DITNC and primary rat astrocytes did not respond to IFNγ, but low levels of NO can be observed after exposure to the three cytokine mixture (Table 1).

We further tested whether rat primary microglial cells (RPM) are capable of responding to cytokines and LPS. Due to difficulty in controlling cell numbers in the RPM preparations, data are based on the amount of proteins in the culture dish. As shown in Figure 5C, stimulation of RPM by cytokines and LPS produced similar levels of NO as compared to that in BV-2 cells.

### Induction of sPLA2-IIA mRNA and protein expression by cytokines and LPS in different glial cell types

**Figure 6:**
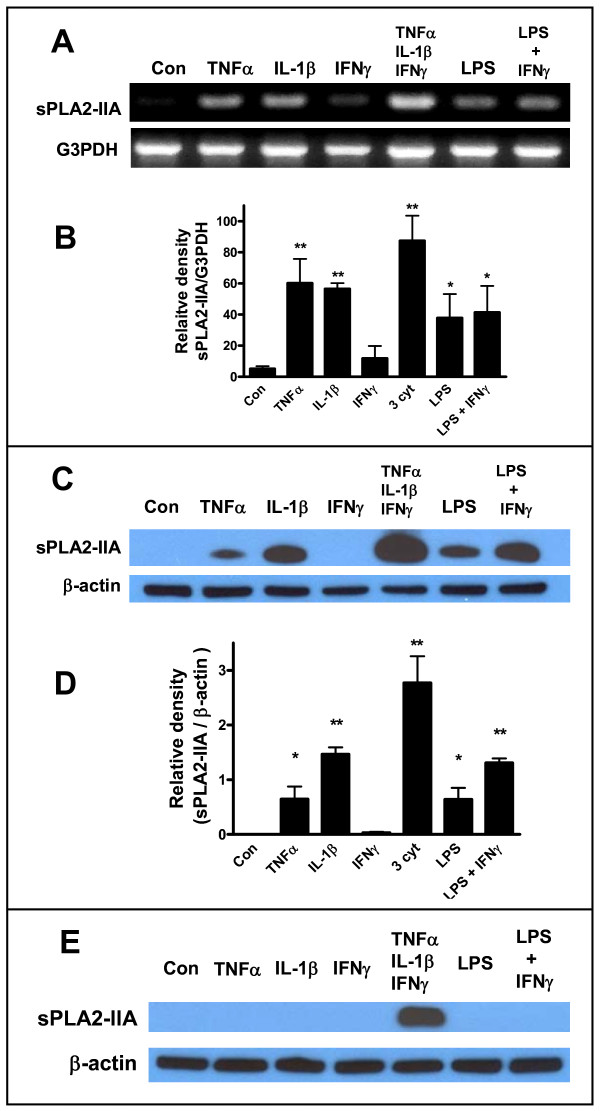
**Cytokines induce sPLA2-IIA mRNA and protein expression in DITNC, and primary astrocytes**. After exposing DITNC astrocytes to TNFα, IL-1β, IFNγ, and LPS for 24 h, sPLA2-IIA mRNA expression was determined by RT-PCR (A), and protein expression was determined by Western blot (C). Quantitative data are expressed as relative density to G3PDH (sPLA2-IIA vs. G3PDH) (B) or to β-actin (sPLA2-IIA vs. β-actin) (D). Results are mean ± SD from three independent experiments (*p < 0.05, **p < 0.01 vs. control, One-way ANOVA, Dunnett's multiple comparison test). (E) Only the three cytokines mixture could cause induction of sPLA2-IIA in primary astrocytes.

In our previous studies, induction of sPLA2-IIA expression by cytokines had been mainly limited to assay of mRNA expression because of lacking suitable antibodies for protein detection [21,22,28]. Furthermore, information about induction of this inflammatory enzyme by microglial cells had also been lacking. In this study, we established a similar pattern for individual cytokines and LPS to induce sPLA2-IIA mRNA and protein expression in DITNC astrocytes. These results clearly indicated the capability for TNFα, IL-1β and LPS, but not IFNγ, to induce sPLA2-IIA mRNA expression (Figures 6A and 6B) and protein expression (Figures 6C and 6D) in DITNC cells. The highest level of expression was observed after treating cells with the three cytokine mixture. However, when primary astrocytes were treated with cytokines and LPS under similar conditions as for DITNC astrocytes, sPLA2-IIA protein expression was observed only after treatment with the three cytokine mixture (Figure 6E).

**Figure 7:**
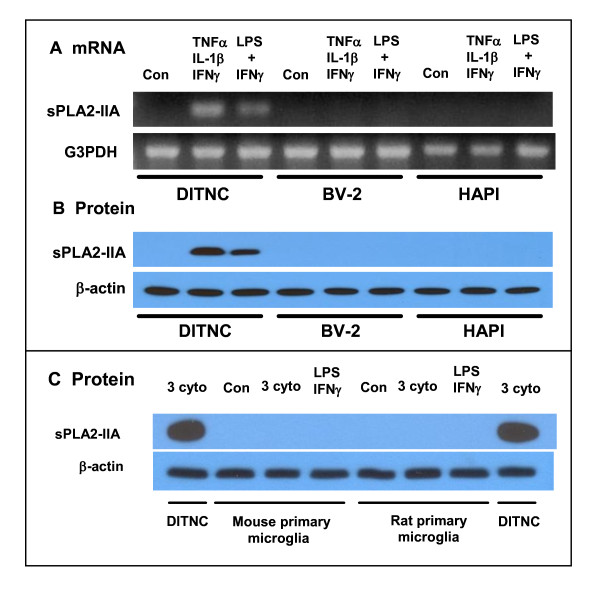
**Cytokines or LPS + IFNγ did not induce sPLA2-IIA in microglial cells**. sPLA2-IIA mRNA (A) and protein (B) were determined by RT-PCR and Western blot in BV-2 and HAPI cells. In this experiment, sPLA2-IIA induced by DITNC astrocytes was used as positive control. (C) Rat primary microglial cells (RPM) did not produce sPLA2-IIA protein after treating with the three cytokine mixture or LPS + IFNγ. Results are from one typical experiment which has been repeated.

We further examined the ability for BV-2 and HAPI cells, as well as primary rat microglial cells, to respond to cytokines and LPS in the induction of sPLA2-IIA mRNA and protein expression. In this study, samples from DITNC astrocytes were used as a positive control. The lack of response in BV-2 cells is expected because these cells are of murine origin. However, it is surprising that cytokines and LPS could not induce sPLA2-IIA mRNA, and protein expression in HAPI cells that are of rat origin (Figure 7A and 7B). In order to further confirm that the lack of response is not due to the immortalization procedure, we tested primary mouse and rat microglial cells and showed that neither cell type could respond to cytokines and LPS to produce sPLA2-IIA (Figure 7C). These results demonstrate that despite the active response to cytokines and LPS in induction of iNOS, microglial cells lack the ability to cause induction of sPLA2-IIA mRNA and protein under cell culture conditions.

### Cytokines and LPS increase sPLA2-IIA immunoreactivity in DITNC and primary astrocytes

**Figure 8:**
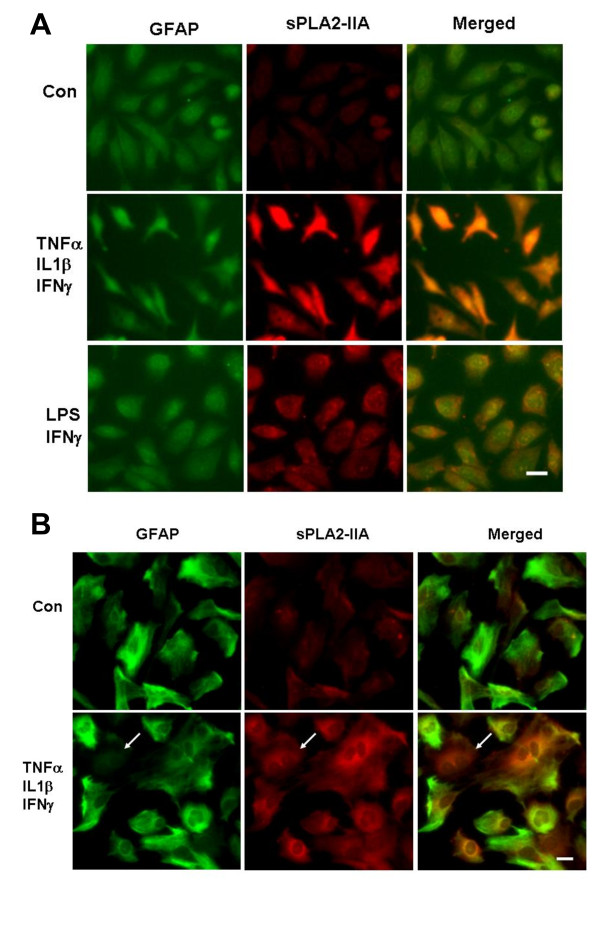
**sPLA2-IIA immunoreactivity in DITNC and primary astrocytes**. (A) DITNC cells were cultured on coverslips and stimulated with cytokines or LPS + IFNγ for 24 h. (B) primary astrocytes were treated with cytokines or LPS + IFNγ for 48 h. After exposure to cytokines and LPS, cells were permeabilized and double-immunostained with GFAP (*left, green*) and sPLA2-IIA (*middle, red*) with merged images (*right*). Scale bar represents 20 μm.

In this study, we have successfully used rabbit polyclonal antibodies against human sPLA2-IIA from BioVendor (Candler, NC) for Western blots, but these antibodies were not suitable for immunocytochemical study. Instead, testing with anti-sPLA2-IIA polyclonal antiserum from Cayman Chemical (Ann Arbor, MI) appeared to give positive immunostaining of sPLA2-IIA in DITNC cells and primary rat astrocytes. As shown in Figure 8A, DITNC cells are positive for GFAP, and an increase in sPLA2-IIA immunoreactivity can be shown upon exposing cells to the three cytokine mixture and LPS + IFNγ for 24 h (Figure 8A). Treatment with primary astrocytes with the three cytokine mixture for 48 h also showed an increase in sPLA2-IIA immunoreactivity (Figure 8B). However, double immunostaining of primary astrocytes with GFAP and sPLA2-IIA indicated variances in GFAP and sPLA2-IIA immunoreactivity after exposure to cytokines. In Figure 8B, we identified a cell (pointed by the white arrow) showing little or none immunoreactivity on GFAP, but substantial staining of sPLA2-IIA. In addition, sPLA2-IIA immunoreactivity appeared to be higher in differentiating cells containing multiple nuclei.

## Discussion

Using immortalized cell lines, we demonstrated substantial differences between microglia (mouse BV-2 and rat HAPI) and astroglia (DITNC) in their responses to pro-inflammatory cytokines and endotoxins. Besides induction of iNOS and sPLA2-IIA, we also examined temporal changes in cell morphology, e.g., formation of filopodia in microglial cells, and upregulation of p-ERK1/2. Thus, information provided by this study is important for selection of cell types as models for testing anti-inflammatory and anti-oxidative compounds on inflammatory responses.

A time course study ranging from 5 min to 4 h indicated that the three cytokines or LPS + IFNγ could induce transient early and late phase increases in p-ERK1/2 expression in BV-2 microglial cells and DITNC astrocytes (Figure 3). The dramatic increase in p-ERK1/2 during 1 to 4 h in BV-2 cells is of particular interest because this increase appears to correlate well with the time for filopodia production (Figure 4A). In agreement with the lack of filopodia production in DITNC astrocytes, these cells did not show a precipitous increase in p-ERK1/2 expression during 1 to 4 h. Studies to further test the induction of filopodia in BV-2 cells by individual cytokines revealed the role of IFNγ and its downstream pathway leading to activation of ERK1/2 (Figure 4D). A study by Nakamura et al. also observed morphological changes in microglial cells upon exposure to LPS [31]. However, our results here provide further evidence of a link between IFNγ and ERK1/2 for induction of filopodia.

IFNγ is known to cause activation of the JAK/STAT pathway, and similar to earlier studies [9], results here demonstrated that IFNγ alone could induce NO production in BV-2 and HAPI cells as well as rat primary microglial cells (data not shown). Besides the interferon regulating factor (IRF-1) and STAT1, transcription factors such as NF-κB are present in the promoter of the iNOS gene [32]. In human macrophages, ERK1/2 activation is critical for phosphorylation of STAT1 induced by IFNγ [29]. The ability for IFNγ alone to induce iNOS in microglial cells is an indication that IFNγ receptor can activate signaling molecules and downstream pathways leading to activation of NF-κB. Our earlier study indicated differences in ERK1/2 activation and temporal changes in PKCδ in the induction of iNOS by IFNγ and LPS [9]. More recently, a study by Jung et al. also indicated IFNγ-induced JAK/STAT and ERK1/2 signaling pathways for expression of iNOS [33].

Data in Table 1 show that under similar treatment conditions with a comparable number of cells plated to the well, BV-2 cells are generally more responsive to cytokines and LPS in the induction of NO as compared to HAPI cells. Based on results in Figure 5C, BV-2 cells are comparable to rat primary microglia in production of NO. Study by Horvath et al. [26] showed low NO production in LPS-stimulated BV-2 cells as compared to primary microglia and HAPI cells. One possible difference is the absence of IFNγ in the study by Horvath et al. [26]. In our study, DITNC and primary rat astrocytes showed considerably lower NO as compared to microglial cells. It is recognized that inflammatory responses in cultured cells can be modified by a number of factors, including the animal source of the cells, culture conditions, seeding density, levels of cytokines and LPS, and time for removal of serum [34]. For example, decreasing serum in culture media could cause morphological changes in HAPI cells [25]. In addition, studies using primary astrocytes need to be particularly cautious about the presence of microglial cells, which may rapidly proliferate upon exposure to cytokines and LPS. In fact, an immunostaining study with primary astroglia/microglia preparations indicated that cytokine-induced iNOS is mainly attributed to microglia and not astrocytes [35]. Our results here showed low but detectable levels of NO upon exposing immortalized (DITNC) and primary astrocytes (after removing microglial cells) to cytokines.

In primary and immortalized astrocytes of rat origin, induction of sPLA2-IIA can be mediated independently by TNFα and IL-1β, without the involvement of IFNγ [21,22,28,36]. Since BV-2 cells are of murine origin, it is not surprising that these cells lack the ability to induce sPLA2-IIA upon exposure to cytokines. However, we were surprised to find that the immortalized HAPI cells, which are of rat origin, also lacked the ability to respond to cytokines and LPS in the induction of sPLA2-IIA (Figure 7A and 7B). Testing with rat primary microglial cells isolated from primary astrocytes further provided data confirming the lack of ability for microglial cells to induce sPLA2-IIA in response to cytokines and LPS (Figure 7C).

In this study, we observed upregulation of sPLA2-IIA immunoreactivity in DITNC astrocytes and in primary astrocytes upon exposure to cytokines and LPS + IFNγ (Figure 8A and 8B). These results are in agreement with observation of sPLA2-IIA in astrocytes in rat brain after focal cerebral ischemic insult [16] and in the Alzheimer brain as compared to age-matched controls [18]. However, double staining with sPLA2-IIA and GFAP in primary astrocytes after exposure to cytokines indicated variances in GFAP and sPLA2-IIA immunoreactivity (Figure 8B). The one cell showing low GFAP but high sPLA2-IIA immunoreactivity suggests that cells other than astrocytes may be present in the primary culture, and that primary astrocytes may undergo different stages of differentiation after exposure to cytokines. Study by Titsworth et al. observed upreguation of sPLA2-IIA in oligodendroglial cells in response to spinal cord injury [20]. Obviously, further studies are needed to investigate mechanism for upregulation of sPLA2-IIA in different glial cell types under in vivo and in vitro conditions.

## Conclusions

This study attempts to lay the ground work for using immortalized glial cells for neuroinflammatory responses, induction of NO and sPLA2-IIA. Our results demonstrated a time-dependent increase in filopodia production upon exposure of microglial cells to IFNγ, and the dependence of ERK1/2 activation for this process. Our results further showed the ability for immortalized microglial cells (BV-2 and HAPI) to produce high levels of NO in response to pro-inflammatory cytokines or LPS while they lack the ability to induce sPLA2-IIA. On the other hand, the immortalized astrocytes (DITNC) proved to be a suitable cell line for studies to elucidate signaling pathways for cytokines to induce sPLA2-IIA expression.

## Competing interests

The authors declare that they have no competing interests.

## Authors' contributions

WS carried out cell culture, treatment with cytokines and LPS, Western blotting, and immunohistochemistry; YZ carried out RT-PCR for mRNA assay; AM helped with the filopodia determination, DA helped to isolate primary microglial cells; JC helped with immunohistochemistry protocol; DH was engaged in the time course study for MTT and NO; JLH helped with preparation of primary astrocytes; AS helped with statistical analysis; ZG helped with microscopy, JH collaborated with providing HAPI cells; AS, AYS, ZG, JH and GAW provided intellectual concepts and help to edit the manuscript; and GYS was involved in design of the project, interpretation of results, and finalizing the manuscript. All authors read and approved the final version of the manuscript.
